# Loss of Response to Long-Term Infliximab Therapy in Children with Crohn’s Disease

**DOI:** 10.3390/ph6101322

**Published:** 2013-10-16

**Authors:** Oliver Gouldthorpe, Anthony G. Catto-Smith, George Alex, Di Simpson

**Affiliations:** 1 Department of Paediatrics, Monash University, Clayton, Victoria 3052, Australia; E-Mail: oliver.gouldthorpe@gmail.com; 2 Department of Gastroenterology, the Royal Children’s Hospital, Parkville, Victoria 3052, Australia; E-Mails: george.alex@rch.org.au (G.A.); di.simpson@rch.org.au (D.S.); 3 Department of Paediatrics, University of Melbourne, Parkville, Victoria 3052, Australia

**Keywords:** Crohn’s disease, paediatrics, infliximab, immunomodulator, growth

## Abstract

Secondary loss of response (LoR) often precludes further use of infliximab in children with Crohn’s disease. Immunomodulators may reduce the incidence of LoR but their combination with infliximab presents safety concerns. We aimed to determine the long-term durability of infliximab response in paediatric Crohn’s, effect of immunomodulators on LoR, and secondarily the effect of infliximab on growth. We retrospectively audited patients on maintenance infliximab at a single centre. Data included height and weight, Paediatric Crohn’s Disease Activity Index (PCDAI), and immunomodulator use. 71 children (32% female, mean age 14.4 years) had been commenced on maintenance infliximab before July 2011. 89% had been on immunomodulators concurrently with infliximab. LoR occurred in 20 (28%), with a median time to LoR of 4.31 years. LoR was significantly increased in children who did not enter remission (PCDAI ≤ 10) after induction (*p* < 0.05). LoR occurred more frequently in the 72% who ceased immunomodulators, but this failed to reach statistical significance (*p* = 0.300). Height and weight SDS improved significantly on infliximab. Infliximab is a durable long-term therapy for paediatric Crohn’s refractory to conventional therapy. A large-magnitude increase in the rate of loss of response after immunomodulator cessation was not observed.

## 1. Introduction

A growing number of children suffer from refractory Crohn’s disease, requiring the use of biological drugs such as infliximab. Biological therapies provide both risks and benefits. Risks in large part relate either to either allergic or infusion related reactions, the risks of infection which are probably less than those related to steroid therapy, and the potential for malignancy. The clear benefit of biologicals lie in their remarkable efficacy for many patients with otherwise treatment-resistant disease [1,2]. Remission may be maintained for long periods on this therapy, but loss of response may occur, leaving children with few other options for a lifetime of disease [3]. There is some uncertainty regarding the incidence of loss of response: clinical trial evidence reports a rate of up to 36% within one year [4], whereas observational evidence suggests a lower incidence, with up to 67% of children continuing infliximab for more than three years [5].

Prolongation of clinical response to infliximab is—on current paediatric evidence—best optimized by strict adherence to scheduled maintenance dosing every eight weeks [4,6]. In adults, the SONIC trial demonstrated a clear benefit of concurrent use of infliximab and azathioprine in reducing the incidence of loss of response [7], but it is not certain that this approach offers any benefit in children. Importantly, subjects in SONIC were naïve to azathioprine, whereas most children who begin infliximab therapy have already failed to respond to immunomodulators [5].

The risks of malignancy—both from treatment and the disease itself—must be weighed carefully in children [7,8]. Young males are at greatest risk of developing the admittedly rare but almost universally fatal hepato-splenic T cell lymphoma when on anti-tumor necrosis factor-α therapy (anti-TNFα) and/or thiopurines such as azathioprine [8]. Meanwhile, it is well accepted that uncontrolled chronic inflammation in Crohn’s disease increases the risk of malignancy [9]. In the absence of clear evidence to support the concurrent use of thiopurines, it is not clear if malignancy risk is lower in disease that is well controlled with infliximab and thiopurines, when compared to less well-controlled disease where exposure to one or both agents is minimized.

Loss of response may represent one of several underlying pathological processes affecting the pharmacology of infliximab. These include immune and non-immune pharmacokinetic effects, and changed pharmacodynamics given the shifting cytokine profile of the underlying disease process over time [3]. Recently, robust adult evidence has emerged regarding the individualization of treatment strategies by identifying the underlying pharmacological process in patients with loss of response [10]. Clinical access to biological therapies for inflammatory bowel disease in children varies internationally [2]. Unfortunately, government funding arrangements in Australia preclude strategies such as dose intensification in children [11].

Growth is an important consideration in children with Crohn’s disease. Growth impairment—weight loss, short stature and/or reduced height velocity—are present at diagnosis in a significant proportion of children [12,13]. In the era before biological therapies such as infliximab, approximately one-fifth of patients achieved a final adult height significantly below their mid-parental expectation [14]. It appears the milieu of cytokines associated with chronic inflammation is dominant in producing growth impairment [15]. For example, TNFα is implicated in the suppression of the growth hormone axis and long bone growth [16,17]. Chronic inflammation may also contribute to combined central and peripheral hypogonadism, with slowed pubertal progression [18]. Correspondingly, infliximab has been observed to increase growth velocity over short periods of therapy [19], but there is little evidence regarding growth benefits during extended periods of therapy.

The major aim of this study was to quantify the long-term incidence of loss of response in children on maintenance infliximab, and to determine if there was a large-magnitude effect of the cessation of immunomodulators on the rate of loss of response. We also aimed to determine the long-term impact of maintenance infliximab therapy on height and weight. Finally, we aimed to establish the characteristics of concurrent adverse events severe enough to warrant hospitalization.

## 2. Experimental Section

### 2.1. Design and Inclusion

We conducted a retrospective case series study of patients of The Royal Children’s Hospital Melbourne. We included only those who had commenced maintenance infliximab for a confirmed diagnosis of Crohn’s disease. We excluded those who were primary non-responders or were otherwise unable to commence maintenance infliximab therapy. The window for inclusion into the study ended on 30 June 2011 for patients commencing infliximab. During maintenance therapy, patients received infliximab targeted to a dose of 5mg/kg every 8 weeks. Ethical approval was granted by the hospital Human Research Ethics Committee.

### 2.2. Data Acquisition

Data were retrieved from hospital records. The initial diagnosis of Crohn’s disease was confirmed, and the Montréal disease classification defined on the basis of endoscopy, histology and medical imaging findings [20,21] immediately prior to commencing infliximab. Age at diagnosis was calculated from the first diagnostic endoscopy. Paediatric Crohn’s Disease Activity Index was recorded prospectively as part of requirements for ongoing drug eligibility under Australian Government funding arrangements [22]. Immunomodulator use was recorded (including azathioprine, 6-mercaptopurine and methotrexate). For patients who were on immunomodulators at baseline but later discontinued them, the date of cessation was recorded.

### 2.3. Outcomes

The primary outcome of the study was secondary loss of response to infliximab. This was defined as a worsening of disease activity in the setting of ongoing infusions, determined by the treating clinician’s global assessment and decision to cease further infusions. Data were censored for subjects who were transferred to adult centres or were still receiving infliximab at the end of the study window; that is, in the case of subjects where loss of response did not occur during their *observed* time on infliximab, data has been analysed accordingly.

Secondary outcomes included adverse events and growth. We defined an adverse event as any event requiring hospital admission. Events were categorised into the following: surgery, exacerbation of disease activity, infection, ICU admission and death. Growth measurements were recorded as height- and weight-for-age standard deviation scores (SDS), and calculated as changes in SDS from baseline.

### 2.4. Statistical Analysis

All calculations were performed using STATA release 11 (StataCorp LP, College Station, TX, USA). Survival analysis methods were employed to examine the rate of loss of response and its associated covariates. The Kaplan-Meier method was used to estimate the duration of ongoing responsiveness to infliximab. The log rank (Mantel-Cox) test and Cox’s proportional hazards regression modelling were used to determine statistical significance. To examine infliximab response beyond significant events, a time-varying covariate method was used. Growth data was interpreted using non-parametric tests of significance. A Wilcoxon signed rank test was used for paired observations, and the Mann-Whitney U test was used for independent samples. We tested for correlation between changes in SDS and other factors using Spearman correlation.

## 3. Results

### 3.1. Study Population

We identified 109 patients who had received infliximab in the period from 2 November 2004 until 30 June 2011. Thirty-eight patients were excluded from the study, consisting of 23 who received only episodic treatment, and 15 who received only a partial induction either because of severe infusion reactions (n = 4), primary non-response (n = 5), or other reasons (n = 6). Of the 71 who commenced maintenance therapy, all had a Paediatric Crohn’s Disease Activity Index (PCDAI) at first infliximab infusion of ≥30. Two cohorts were identified based on age at commencement of infliximab. Government funding for maintenance infliximab became available from 2007, and time from onset of disease to commencement of infliximab was shorter in this group than in those who commenced infliximab before 2007 (Table 1). The median period of observation was 1.54 years (range: 0.29–5.75). The total period of observation was 141.5 patient-years.

**Table 1:** Baseline Characteristics.

Demographics			
Female	23 (32.4%)		
Age (years)	*14.4 (3.95–20.1)*		
**History of disease**			
Duration (weeks)	Pre-2007	2007-Onwards	
	*135 (12–578)*	*54 (5–128)*	*p* < 0.001
**Disease classification (Montréal)**			
Age at Diagnosis	<16 years	>16 years	
	65 (91.5%)	6 (8.45%)	
Location	Ileocolonic	Colonic	Ileal
	45 (63.4%)	22 (31.0%)	4 (5.63%)
+ upper GI	41 (57.7%)		
Behaviour	Inflammatory	Fibrostenotic	Penetrating
	60 (83%)	4 (7%)	7 (10%)
+ perianal	28 (39.4%)		
**Disease severity (PCDAI** †; n = 59)			
Severe	33 (55.9%)	PCDAI ≥ 40	
Moderate	26 (44.1%)	PCDAI 30–37.5	
**Medications**			
IM *	Thiopurines	Methotrexate	None
	46 (64.8%)	10 (14.1%)	15 (21.1%)
Corticosteroids	≥1 mg/kg	<1 mg/kg	None
	3 (4.23%)	10 (14.1%)	58 (81.7%)
Budesonide	3 (4.23%)		

Values given as number of subjects—n (%) or as a *median (min.–max.)*; ^†^ Paediatric Crohn’s Disease Activity Index; * immunomodulator.

### 3.2. Loss of Response

**Figure 1:**
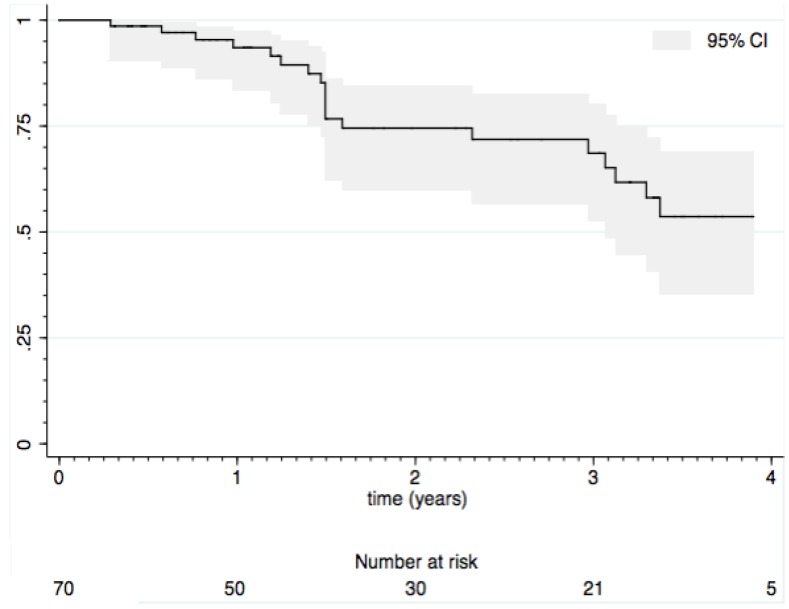
Duration of response to maintenance infliximab (Kaplan-Meier estimate).

Twenty subjects ceased infliximab infusions during the study. In all cases, the indication for cessation was secondary loss of response. This represented 0.14 events per person-year of exposure (Figure 1).

**Figure 2:**
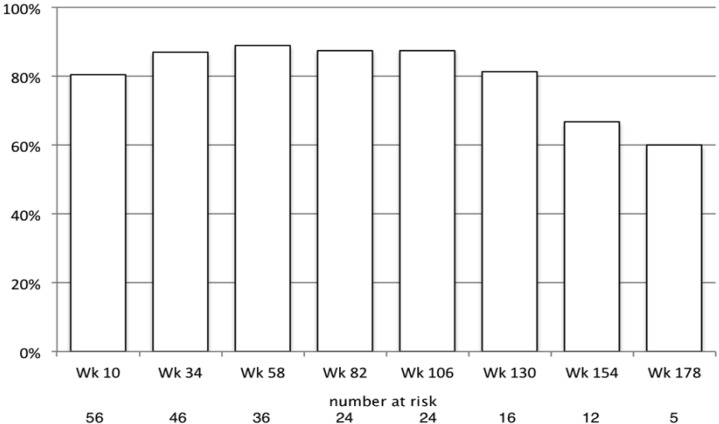
Proportion in remission while continuing infliximab (PCDAI ≤ 10).

Complete PCDAI values were available for 56 of the 71 subjects (78.9%). At Week 10 after the first induction infliximab infusion, forty-five (80.4%) had entered remission (PCDAI ≤ 10); ten of the remaining patients had mild disease activity (PCDAI >10 but <30), and one had moderate disease activity (PCDAI ≥ 30). All had responded to infliximab as defined by a PCDAI reduction of at least 12.5 points. Between week 34 and week 130, 80%–89% of patients who were still on infliximab were in remission. At Week 154, eight of twelve patients who were still on infliximab were in remission; at Week 178, three of five patients who were still on infliximab were in remission (Figure 2).

Thirteen subjects (18%) were on oral corticosteroids at the time of commencing infliximab induction. After ten weeks, there had been a statistically significant reduction in the per kilogram dose of corticosteroids (*p* = 0.0192), with most subjects (n = 10) having been completely weaned. Three additional subjects who were on enteral-release budesonide at baseline had also ceased budesonide by ten weeks.

Using a multivariate proportional hazards regression model (Table 2), we examined factors that might be associated with secondary loss of response. We found that patients who entered remission at Week 10 had a statistically significant reduction in the rate of secondary loss of response, when compared to those who had not (*p* = 0.027). Ileal disease location appeared to increase the risk of secondary loss of response, but this failed to reach statistical significance.

### 3.3. Impact of Immunomodulators

**Figure 3:**
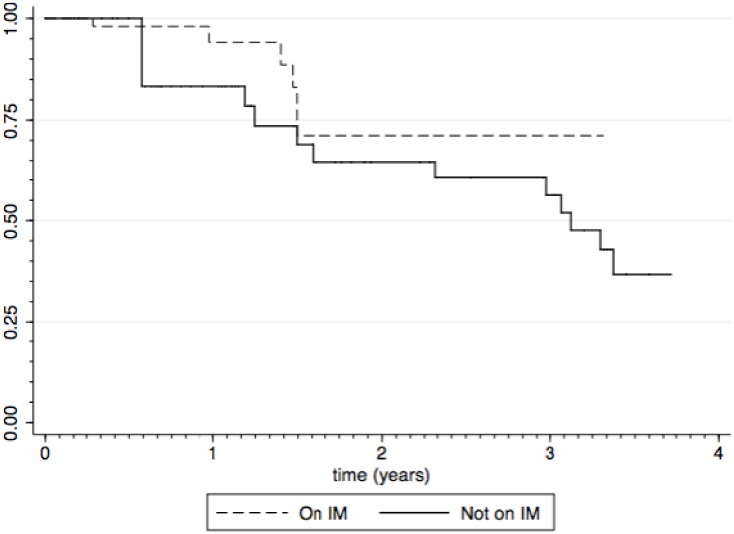
Duration of response to infliximab by immunomodulators (IM) use (Kaplan-Meier estimate).

Fifty-six subjects (79%) were on immunomodulator therapy at baseline, and thirty-nine ceased this during the study. The median time to immunomodulator cessation was 42 weeks (range: 9–300). On a categorical basis, there was no statistically significant difference in the rate of loss of response between those who had immunomodulator therapy of any duration compared with those who did not (*p* = 0.130). On time-varying covariate analysis there was a trend towards an increase in loss of response following cessation of immunomodulator therapy, however this did not reach statistical significance (*p* = 0.300); Figure 3.

**Table 2:** Secondary loss of response to infliximab and clinical factors. Proportional hazards regression model.

Variable	Coefficient	Std. error	*p* value
Remission post induction	−1.31	0.593	0.027
Ileal location	1.68	0.906	0.064
Induction age	62.0	42.4	0.144
Disease duration	−1.19	0.814	0.144
Diagnosis age	−61.9	42.4	0.145
Post-IM status	0.668	0.618	0.280
Female gender	0.069	0.770	0.929

### 3.4. Growth

Growth data were available for 67 subjects (94% of total subjects) at baseline. Between diagnosis and commencing infliximab (baseline), there had been a significant fall in median height-for-age SDS of −0.238 (*p* < 0.001). At the time of the first induction dose of infliximab, the median height-for-age SDS was −0.329 and the median weight-for-age SDS was −0.772. We identified no statistically significant differences in baseline height or weight-for-age SDS on the basis of gender, disease behaviour, disease location, or disease duration.

After 34 weeks, there had been a significant increase in median height-for-age SDS of +0.0576 (*p* = 0.0469). The increase in median weight-for-age SDS was +0.518 (*p* < 0.001). At this time point only, those with fistulising disease experienced a greater improvement in median height-for-age SDS compared with those without fistulising disease (+0.453 and +0.0745 respectively; *p* = 0.0326). Also at this time point only, we identified greater median improvements in weight-for-age SDS amongst those not on immunomodulators (0.109 *vs.* 0.0414; *p* = 0.0502). We found no other variables that were associated with differential improvements in height or weight at any other time point, including gender, disease location, immunomodulator use, baseline prednisolone use, and disease remission after induction.

Whilst on infliximab, the majority experienced continuous, significant improvements in height SDS over baseline (*p* < 0.05; Table 3).

**Table 3:** Median height and weight-for-age standard deviation scores after commencing infliximab observations (* *p* < 0.05 *vs.* baseline).

	Diagnosis	Baseline	Change from baseline (weeks on infliximab)					
			34	58	82	106	130	154
**n**	41	67	51	43	36	29	20	16
**Height SDS**	−0.061	−0.33	0.058 *	0.32 *	0.40 *	0.65 *	0.74 *	0.86 *
**Weight SDS**	NA	−0.77	0.52 *	0.62 *	0.46 *	0.61 *	0.69 *	0.48 *

Among all 67 patients, regardless of time spent on maintenance infliximab, the ultimate median change in height SDS was +0.243 (*p* < 0.001). There was no significant correlation between time on infliximab and ultimate improvement (Spearman’s rho = 0.24 *p* = 0.0826). There was a moderate negative correlation between baseline height SDS and final height SDS (Spearman’s rho = −0.4087; *p* = 0.0022).

### 3.5. Adverse Events

Nineteen adverse events were recorded during the study in 15 patients (Appendix A). Events occurred at a rate of 0.13 events per person-year of exposure. On Kaplan-Meier analysis, 21.2% of subjects are expected to have at least one adverse event within two years (95% confidence interval: 13.0%–35.0%). We could not identify categorical covariates that had a statistically significant effect on the rate of adverse events. There was no significant relationship between immunomodulator exposure during infliximab therapy and the development of any adverse events.

Three patients had a loss of response to infliximab that resulted in an exacerbation requiring hospital admission (thus simultaneously also meeting our criterion for an adverse outcome). Of twelve subjects who remained on infliximab after their first adverse event, all subsequently continued in remission for the period of the study. The Kaplan-Meier estimate of median continuation on infliximab for the study period after an adverse event was 1.59 years.

There were seven exacerbations of Crohn’s disease requiring hospital admission among six patients. All required the administration of corticosteroids or antibiotics in addition to other medical therapies. Events occurred at a rate of 0.0465 events per person-year of exposure.

Six subjects received surgery during the study period (0.0422 events per person-year). Perianal surgery was undertaken in four patients and abdominal surgery with local resection in two.

Six patients experienced one infection each. Four cases were of pneumonia. In all cases, the aetiological agent was not identified. All were treated with empirical antibiotics, and had an uncomplicated recovery. Two cases of herpes zoster were identified, but did not require hospital admission. Both were treated with supportive therapies, and made uncomplicated recoveries. There were no deaths, malignancies or ICU admissions among subjects during the study period.

## 4. Discussion

We aimed to describe the long-term durability of clinical response during maintenance infliximab therapy for children with Crohn’s disease. We estimated the likelihood of continuing clinical response to infliximab as 74% and 54% at two and four years, respectively. There was a high rate of remission and very significant reduction in the use of corticosteroids. Those who entered remission after induction were significantly less likely to experience loss of response. Discontinuation of maintenance treatment occurred exclusively because of secondary loss of response.

There was a suspicion that the ongoing clinical response to infliximab appeared to be better where immunomodulators were being used concurrently, though this failed to achieve statistical significance. However this type of retrospective study is unlikely to answer this question. Only a small number of patients were not receiving immunomodulators at induction with infliximab in our study. In contrast, of those who were receiving immunomodulators, more than half had ceased these within a year. We found no difference in durability of response to infliximab in either of these two situations. The evidence for added benefit from concurrent immunomodulators remains equivocal but most clinicians appear uncomfortable with dispensing with their use, particularly in the first 6 months of infliximab therapy.

In the REACH trial [4], all subjects were required to be on an immunomodulators for the first 8 weeks after commencing infliximab but only 63% were still showing a clinical response on 8 weekly infliximab infusions after one year. This compares to 80% in our study. However it is not clear from the REACH study how many patients were still on immunomodulators at week 54.

Hyams *et al.* [5], found similar rates of long-term response to infliximab as we found in our study (93% at 1 year, 78% at 2 years and 67% at 3 years in 128 patients), but with a substantially greater long term use of immunomodulators than in our study—75% of participants remained on immunomodulators after three years of infliximab therapy. In contrast, only 3% of patients in our study were on immunomodulators after three years but with a similar remission rate to that of Hyams *et al.* In adults, van Assche *et al.* [23] found no significant difference in the rate of loss of response between those ceasing immunomodulators after 6 months and those continuing, when evaluated after 2 years. However, only approximately 40% had not developed a loss of response in either group by this point.

We did not have PCDAI measurements at the time of infliximab cessation. However, it has been demonstrated that physicians are able to reliably make valid assessments of disease activity, and it was on this basis that we defined secondary loss of response [24]. We recorded loss of response at the time of eventual infliximab cessation. This may have distorted our assessment of time to loss of response, as subjects may have lost response before discontinuing infusions, but the regulatory authorities in Australia require patients to continue to show clinical response in order to qualify for ongoing federally-funded infliximab.

Loss of response can be addressed by altering dosage or dose intervals, but this option is not easily available in Australia through current government funding mechanisms. The paucity of options for managing loss of response emphasises the importance of its prevention. The RESEAT observational study reported outcomes in 115 children who were treated with adalimumab [25]. Prior loss of response or adverse reaction to infliximab had occurred in 92% of subjects. By one year, the rate of clinical remission on adalimumab was only 42%. Similarly disappointing rates of remission (41% at one year in 72 patients of whom 94% had received prior infliximab) have been reported in a survey of UK and Ireland clinicians [26].

Infliximab had a substantial beneficial impact on growth in our study. Most suggest that infliximab indirectly improves growth through a permissive effect on pubertal progression [19,27]. In the REACH study, growth velocity was improved in children who had a significantly delayed skeletal maturity [4]. Walters *et al.* observed significant increases in height-for-age only among those in early pubertal stages (Tanner I-III) [19]. Malik *et al.* observed that while significant improvements in height-for-age occurred over a twelve-month period, this included those who remained pre-pubescent, suggesting that infliximab also has a direct impact on growth, independent of its effects on pubertal progression [27]. Unfortunately we did not have records of Tanner staging or radiological bone age that would have enabled us to comment on pubertal progression in this group.

The natural history of paediatric Crohn’s disease is progressive, resulting in a significant cumulative incidence of surgery. Prospective cohort studies of children show that 20%–34% have their first surgery within five years of diagnosis [28,29]. It is difficult to directly compare patients from these cohorts, as they include children with less severe disease. However, the fact that sixty-five children in our study with moderate to severe disease prior to starting infliximab did not undergo any surgery is likely to represent a significant beneficial effect, compared with now historical groups who did not receive infliximab. 50% of surgery occurred in children who already had complicated disease behaviour at baseline. This suggests that very few subjects had progression of disease behaviour, again contrasting with the expected natural history of disease.

## 5. Conclusions

The availability of biological agents such as infliximab has transformed the care of patients with Crohn’s disease and children in particular. This study has demonstrated the efficacy of infliximab in ordinary clinical practice for the long-term management of this life-long illness. Moderate to severe disease is generally well controlled and growth is improved, but the biggest single potential problem is loss of response to these extremely effective drugs. Immunomodulators may reduce the risk of loss of response but this is difficult to confirm in children. Immunomodulators carry with them the potential to contribute to malignancy, but it may be that the difficult-to-quantify benefit of reducing risk of loss of response outweighs the extremely small risk of malignancy. Assessment of individual risk is a difficult and emotive subject and it can be hard for many patients and families to be objective. Striking a balance between benefits and risks should be considered on a case-by-case basis, and involve open and honest discussions among clinicians, patients and their families.

## Abbreviations

IFX: Infliximab
IM: Immunomodulator
LoR: Loss of Response (to infliximab)
PCDAI: Paediatric Crohn’s disease activity index
REACH: A Randomized; multicenter; open-label study to Evaluate the safety and efficacy of Anti-TNF-α Chimeric monoclonal antibody in pediatric subjects with moderate to severe Crohn’s disease
SDS: Standard Deviation Score
SONIC: Study of Immunomodulator Naive Patients in Crohn’s Disease
TNF: Tumor Necrosis Factor

## Appendix A

**Table:** Clinical details of adverse events.

ID	Type	Clinical information	Time on IFX at event	Total time on IFX	Secondary loss of response
10	Surgery	Drainage of perianal abscess	0.851	1.40	Yes
12	Infection	Pneumonia (community-acquired)	0.175	0.767	Yes
30	Infection	Herpes zoster	2.38	3.49	No
31	Surgery	Ileocaecal resection	0.104	2.53	No
31	Exacerbation	IV steroids	0.350	2.53	No
36	Surgery	Drainage of perianal abscess with seton placement	0.361	2.57	No
51	Exacerbation	IV steroids	0.329	0.594	No
54	Infection	Pneumonia (community-acquired)	1.13	3.12	Yes
56	Surgery	Sigmoid dilatation complicated by perforation	0.638	3.35	No
64	Infection	Pneumonia (community acquired)	0.222	1.25	Yes
67	Surgery	Drainage of perianal abscess	1.49	1.49	Yes
68	Surgery	Drainage of perianal Abscess	1.59	1.59	Yes
68	Exacerbation	IV antibiotics	0.704	1.59	Yes
68	Infection	Pneumonia (community acquired)	1.28	1.59	Yes
75	Exacerbation	IV steroids	0.287	0.287	Yes
86	Exacerbation	IV steroids	2.32	2.32	Yes
87	Infection	Herpes zoster	1.10	1.49	Yes
95	Exacerbation (Erythema nodosum, episcleritis)	IV steroids	0.137	3.20	No
95	Exacerbation	IV steroids	1.57	3.20	No

## References

[1] Yang L.S., Alex G., Catto-Smith A.G. (2012). The use of biological agents in pediatric inflammatory bowel disease. Curr. Opin. Peds..

[2] Gouldthorpe O., Catto-Smith A.G., Alex G. (2011). Biologics in paediatric Crohn’s Disease. Gastro Res. Prac.

[3] Yanai H., Hanauer S.B. (2011). Assessing Response and Loss of Response to Biological Therapies in IBD. Am. J. Gastroenterol..

[4] Hyams J., Crandall W., Kugathasan S., Griffiths A., Olson A., Johanns J., Liu G., Travers S., Heuschkel R., Markowitz J. (2007). Induction and Maintenance Infliximab Therapy for the Treatment of Moderate-to-Severe Crohn’s Disease in Children. Gastroenterology.

[5] Hyams J.S., Lerer T., Griffiths A., Pfefferkorn M., Kugathasan S., Evans J., Otley A., Carvalho R., Mack D., Bousvaros A. (2009). Long-term outcome of maintenance infliximab therapy in children with Crohn’s disease. Inflamm. Bowel Dis..

[6] Ruemmele F.M., Lachaux A., Cézard J.P., Morali A., Maurage C., Giniès J.L., Viola S., Goulet O., Lamireau T., Scaillon M. (2009). Efficacy of infliximab in pediatric Crohn’s disease: A randomized multicenter open-label trial comparing scheduled to on demand maintenance therapy. Inflamm. Bowel Dis..

[7] Colombel J.F., Sandborn W.J., Reinisch W., Mantzaris G.J., Kornbluth A., Rachmilewitz D., Lichtiger S., D’Haens G., Diamond R.H., Broussard D.L. (2010). Infliximab, azathioprine, or combination therapy for Crohn’s disease. N. Engl. J. Med..

[8] Kotlyar D.S., Osterman M.T., Diamond R.H., Porter D., Blonski W.C., Wasik M., Sampat S., Mendizabal M., Lin M.V., Lichtenstein G.R. (2011). A systematic review of factors that contribute to Hepatosplenic T-Cell Lymphoma in patients with Inflammatory Bowel Disease. Clin. Gastroenterol. Hepatol..

[9] Ullman T.A., Itzkowitz S.H. (2011). Intestinal inflammation and cancer. Gastroenterology.

[10] Steenholdt C., Brynskov J., Thomsen O.O., Munck L.K., Fallingborg J., Christensen L.A., Pedersen G., Kjeldsen J., Jacobsen B.A., Oxholm A.S. (2013). Individualised therapy is more cost-effective than dose intensification in patients with Crohn’s disease who lose response to anti-TNF treatment: A randomised, controlled trial. Gut.

[11] Connell W., Andrews J.M., Brown S., Sparrow M. (2010). Practical guidelines for treating inflammatory bowel disease safely with anti-tumour necrosis factor therapy in Australia. Intern. Med. J..

[12] Motil K.J., Grand R.J., Davis-Kraft L., Ferlic L.L., Smith E.O. (1993). Growth failure in children with inflammatory bowel disease: A prospective study. Gastroenterology.

[13] Sawczenko A., Sandhu B.K. (2003). Presenting features of inflammatory bowel disease in Great Britain and Ireland. Arch. Dis. Child..

[14] Sawczenko A., Ballinger A.B., Savage M.O., Sanderson I.R. (2006). Clinical features affecting final adult height in patients with pediatric-onset Crohn’s disease. Pediatrics.

[15] MacRae V.E., Wong S.C., Farquharson C., Ahmed S.F. (2006). Cytokine actions in growth disorders associated with pediatric chronic inflammatory diseases (review). Int. J. Mol. Med..

[16] Wong S.C., Smyth A., McNeill E., Galloway P.J., Hassan K., McGrogan P., Ahmed S.F. (2010). The growth hormone insulin-like growth factor 1 axis in children and adolescents with inflammatory bowel disease and growth retardation. Clin. Endocrinol. (Oxf.).

[17] MacRae V.E., Farquharson C., Ahmed S.F. (2006). The restricted potential for recovery of growth plate chondrogenesis and longitudinal bone growth following exposure to pro-inflammatory cytokines. J. Endocrinol..

[18] Pfefferkorn M., Burke G., Griffiths A., Markowitz J., Rosh J., Mack D., Otley A., Kugathasan S., Evans J., Bousvaros A. (2009). Growth abnormalities persist in newly diagnosed children with Crohn disease despite current treatment paradigms. J. Pediatr. Gastroenterol. Nutr..

[19] Walters T.D., Gilman A.R., Griffiths A.M. (2007). Linear growth improves during infliximab therapy in children with chronically active severe Crohn’s disease. Inflamm. Bowel Dis..

[20] Van Assche G., Dignass A., Panes J., Beaugerie L., Karagiannis J., Allez M., Ochsenkuhn T., Orchard T., Rogler G., Louis E. (2010). The second European evidence-based Consensus on the diagnosis and management of Crohn’s disease: Definitions and diagnosis. J. Crohns. Colitis.

[21] Satsangi J., Silverberg M.S., Vermeire S., Colombel J.F. (2006). The Montreal classification of inflammatory bowel disease: Controversies, consensus, and implications. Gut.

[22] http://pbs.gov.au/medicine/item/4284L-5753T-5754W-5755X-5756Y-5757B-5758C-6397Q-6448J-6496X-9612X-9613Y-9617E-9654D-9674E.

[23] Van Assche G., Magdelaine-Beuzelin C., D’Haens G., Baert F., Noman M., Vermeire S., Ternant D., Watier H., Paintaud G., Rutgeerts P. (2008). Withdrawal of immunosuppression in Crohn’s disease treated with scheduled infliximab maintenance: A randomized trial. Gastroenterology.

[24] Turner D., Griffiths A.M., Walters T.D., Seah T., Markowitz J., Pfefferkorn M., Keljo D., Otley A., Leleiko N.S., Mack D. (2010). Appraisal of the pediatric Crohn’s disease activity index on four prospectively collected datasets: recommended cutoff values and clinimetric properties. Am. J. Gastroenterol..

[25] Rosh J.R., Lerer T., Markowitz J., Goli S.R., Mamula P., Noe J.D., Pfefferkorn M.D., Kelleher K.T., Griffiths A.M., Kugathasan S. (2009). Retrospective evaluation of the safety and effect of adalimumab therapy (RESEAT) in pediatric Crohn’s disease. Am. J. Gastroenterol..

[26] Russell R.K., Wilson M.L., Loganathan S., Bourke B., Kiparissi F., Mahdi G., Torrente F., Rodrigues A., Davies I., Thomas A. (2011). A British Society of Paediatric Gastroenterology, Hepatology and Nutrition survey of the effectiveness and safety of adalimumab in children with inflammatory bowel disease. Aliment. Pharmacol. Ther..

[27] Malik S., Wong S., Bishop J., Hassan K., McGrogan P., Ahmed S., Russell R. (2011). Improvement in growth of children with crohn disease following anti-TNF-α therapy can be independent of pubertal progress and glucocorticoid reduction. J. Pediatr. Gastroenterol. Nutr..

[28] Van Limbergen J., Russell R.K., Drummond H.E., Aldhous M.C., Round N.K., Nimmo E.R., Smith L., Gillett P.M., McGrogan P., Weaver L.T. (2008). Definition of phenotypic characteristics of childhood-onset inflammatory bowel disease. Gastroenterology.

[29] Vernier-Massouille G., Balde M., Salleron J., Turck D., Dupas J.L., Mouterde O., Merle V., Salomez J.L., Branche J., Marti R. (2008). Natural history of pediatric Crohn’s disease: A population-based cohort study. Gastroenterology.

